# Genotype-by-environment interaction with high-dimensional environmental data: an example in pigs

**DOI:** 10.1186/s12711-025-00974-2

**Published:** 2025-06-05

**Authors:** Fernando Bussiman, Daniela Lourenco, Jorge Hidalgo, Ching-Yi Chen, Justin Holl, Ignacy Misztal, Zulma G. Vitezica

**Affiliations:** 1 Department of Animal and Dairy Science, University of Georgia, Athens, GA 30602 USA; 2 Genus Pig Improvement Company, Hendersonville, TN 37075 USA; 3 GenPhySE, INPT, INRAE, ENVT, 31326 Castanet Tolosan, France

## Abstract

**Background:**

In traditional genetic prediction models, environments are typically treated as uncorrelated effects, either fixed or random. Environments can be correlated when they share the same location, management practices, or climate conditions. The temperature-humidity index (THI) is often used to address environmental effects related to climate or heat stress. However, it does not fully describe the complete climate profile of a specific location. Therefore, it is more appropriate to use multiple environmental covariates (ECs), when available, to describe the weather in a specific environment. This raises the question of whether publicly available weather information (such as NASA POWER) is useful for genomic predictions. Genotype-by-environment interaction (GxE) can be modeled using multiple-trait models or reaction norms. However, the former requires a substantial number of records per environment, while the latter can result in over-parametrized models when the number of ECs is large. This study investigated whether using ECs is a suitable strategy to correlate environments (herds) and to model GxE in the genomic prediction of purebred pigs for production traits.

**Results:**

We evaluated different models to account for environmental effects and GxE. When environments were correlated based on ECs, we observed an increase in environmental variance, which was accompanied by an increase in phenotypic variance and a decrease in heritability. Furthermore, including environments as an uncorrelated random effect yielded the same accuracy of estimated breeding values as treating them as correlated based on weather information. All the tested models exhibited the same bias, but the predictions from the multiple-trait models were under-dispersed. Evidence of GxE was observed for both traits; however, there were more genetically unconnected environments for backfat thickness than for average daily gain.

**Conclusions:**

Using outdoor weather information to correlate environments and model GxE offers limited advantages for genomic predictions in pigs. Although it adds complexity to the model and increases computing time without improving accuracy, it does enhance model fit. Including environment information (e.g. herd effect) as an uncorrelated random effect in the model could help address GxE and environmental effects.

**Supplementary Information:**

The online version contains supplementary material available at 10.1186/s12711-025-00974-2.

## Background

Fluctuations in environmental variables and increasing temperatures due to climate change pose a significant concern for livestock production [1]. These seasonal impacts may be seen as heat-stress effects, which are evident in several species worldwide. They have a detrimental impact on animal welfare and disease incidence, causing physiological changes [2]. The more challenging the environment, the greater the negative effects on the productivity, performance, growth, and development of animals exposed to such conditions [3].

Pigs are particularly susceptible to heat stress due to a lack of physiological adaptation, as pigs’ sweat glands do not respond to heat stress [4], and they can only dissipate up to 50% of their heat production by respiratory evaporation [5]. As a result, even with enhanced cooling strategies in pig farms, production losses associated with heat stress remain a concern [6, 7]. Biologically, with high-production levels, pigs exhibit an increase in heat production, although the thermoregulatory mechanism behind this still needs to be fully understood [8].

Environmental effects are traditionally accounted for by fitting herd as an effect in the prediction model [1] and/or by incorporating environmental effects into contemporary groups (CGs) [9]. While some researchers advocate for modeling these effects as random [10, 11], fitting them as fixed effects remains more common [11, 12]. In a fixed effects modeling framework, herds are assumed to be completely independent with no shared characteristics. However, Chauan and Thompson proposed treating CGs as a random effect, allowing for a covariance among them [10]. When two herds are physically close, they are expected to share similarities in climate, geographical, and management conditions [1, 13]. Thus, when different environments experience the same weather conditions, one can expect an additional layer of similarity among these environments.

In livestock, correlated environmental effects have been studied through various approaches and environmental descriptors, aiming to enhance the reliability of genetic predictions. For example, Tiezzi et al. utilized geographical, management, climate, and CG information to model environmental (co)variance structures in US Holstein cattle [14]. Cuyabano et al. employed global positioning system coordinates (GPS) to correlate herds in the genomic evaluation of Hanwoo beef cattle [13]. Similarly, Makanjuola et al. applied the temperature-humidity index (THI) to the model covariance structure among herds for the same breed [1]. Selle et al. analyzed spatial relationships between herds to account for environmental effects in smallholder dairy cattle [15]. Lastly, Santana et al. used a Gaussian kernel based on geographical coordinates to model herd effects in beef cattle [16].

Traditionally, the environmental effects associated with climate or heat stress are measured using some function of the THI [1, 17–19]. However, evidence suggests that the THI is not the most effective method for accounting for these effects, as THI calculations must be tailored to the region in which they are implemented [20], and even then, temperature often serves just as well at predicting heat stress as the THI [21, 22]. Generally, genetic evaluations that account for heat stress involve finding a THI threshold above which the phenotype begins to decline. This process leads to calculating the heat load (HL) as a function of THI, followed by fitting a single reaction norm model of HL for genetic evaluation [17–20]. Employing multiple environmental covariates ECs could result in a more flexible approach, facilitating a better characterization of the environment. However, if a reaction-norm (or random regression) model is used, a large number of ECs can super-parameterize the model.

Accounting for ECs also allows for modeling the genotype-by-environment interaction (GxE) based on climate effects. Usually, GxE is modeled either using a multiple-trait or a reaction-norm model [23]. The first approach considers records from different environments as distinct traits and assumes a genetic correlation among them [17, 24–26]. The second approach requires a continuous environmental gradient and models estimated breeding values (EBV) as a function of the ECs [17–19]. Conversely, Lopez-Cruz et al. proposed a third approach that fits two genetic effects in the multiple-trait model [27]: one across environments and another specific to each environment. However this does not allow for ECs to be considered to avoid double-counting environmental effects. Recently, Zhao et al. derived a similar model to study quantitative trait loci-by-environment interactions [28].

Jarquín et al. proposed modeling GxE using covariance functions [29]. This approach correlates different environments based on the ECs, and the covariance between the EBV and the environmental effects accounts for the GxE [30]. The main advantage here is that modeling the ECs and GxE through covariance functions alleviates the problem of super-parametrization and provides a better characterization of the environmental conditions. Moreover, as the dimension of the covariance among environments is fixed (number of environments^2^), one can fit daily ECs, allowing for a high-dimensional reaction norm. Thus, this study aimed to evaluate the validity of high-dimensional environmental data in modeling correlated herd effects and GxE through covariance functions, using genomic prediction of production traits in purebred pigs as a case study.

## Methods

### Phenotypic, genomic, and environmental data

Data were provided by the Pig Improvement Company (PIC: a Genus company, Hendersonville, TN, USA). Approval from the Animal Care and Use Committee was not required for this study as the data were obtained from an existing database. The original data set comprises 95,873 genotyped and phenotyped animals. The original dataset was down-sampled to enable variance component estimation with genomic information. We ensured the sample was representative of the whole dataset by implementing the following process: first, we set the minimum number of genotyped animals (N = 30,000). Next, the phenotypic dataset was refined to remove outliers (i.e. records deviating ± 3 standard deviations from the phenotypic mean) and CGs (defined later) that lacked phenotypic variation or that had fewer than 30 records. Subsequently, the number of animals to be sampled (s) was calculated as the next integer greater than N/mCG (where N = 30,000 and mCG is the average number of animals per contemporary group). The final dataset analyzed consisted of 35,596 records for average daily gain (ADG) and 31,105 for backfat thickness (BFT) from purebred pigs born between 2009 and 2020, genotyped and phenotyped. ADG and BFT were measured at the end of a growth performance testing period, which began at approximately 10 weeks of age and ended at around 20 weeks of age. The ADG was calculated by dividing weight by age, and BFT was measured using ultrasound. Table 1 presents the descriptive statistics for the dataset used. Records were collected from 11 farms, all with the same health status, located throughout the northern hemisphere. The distribution of animals per farm is shown in Table 2.

**Table 1:** Descriptive statistics for each trait in the dataset

Statistic	Trait	
	ADG (g)	BFT (mm)
N^1^	35,597	32,105
Mean	751.29	8.06
SD	97.46	1.97
Min	450.70	5.00
Max	1000.00	18.23
YOB^2^	2009–2020	2009–2020
NCG^3^	203	220
NE^4^	11	11

*ADG* average daily gain, *BFT* backfat thickness

^1^Number of records

^2^Birth year range

^3^Number of contemporary groups

^4^Number of environments (farms)

**Table 2:** Number of records per trait for each environment (farm), along with their location (country)

Environment	Trait		Location
	ADG	BFT	Country
4	5321	5185	Canada
6	3072	2585	Canada
2	674	284	United States
5	382	234	United States
11	3684	3251	United States
8	11,924	10,181	United States
7	6721	6839	United States
1	122	186	Czech Republic
3	213	192	Germany
9	1017	1205	Ireland
10	2467	1963	Ireland

*ADG* average daily gain, *BFT* backfat thickness

Genotypes were imputed up to 50 K single nucleotide polymorphisms (SNPs). After quality control using default parameters (excluding SNPs with minor allele frequency < 0.01, call rate < 0.90, monomorphic, Hardy–Weinberg Equilibrium with a maximum difference between observed and expected heterozygosity > 0.15, number of Mendelian conflicts > 1%) by preGSf90 [31], 44,368 autosomal SNPs remained, which were used to build genomic relationship matrices. All animals used in the analyses were phenotyped and genotyped. Therefore, genetic relationships among animals across environments were implicitly modeled through the genomic relationship matrix, which connects all animals across all environments.

#### Environmental covariates

**Fig. 1:**
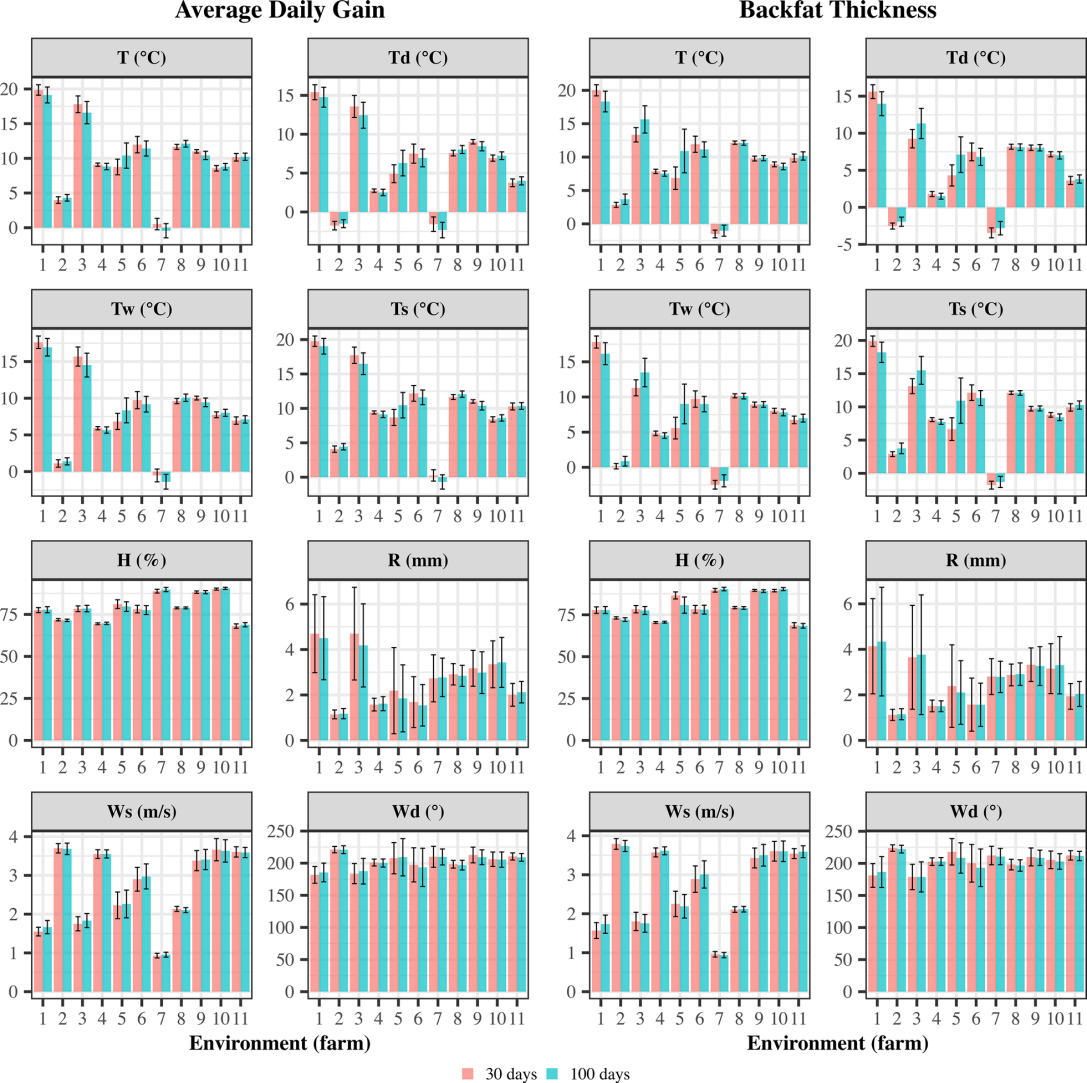
Averages and standard deviations of environmental covariates within each environment (farm) for 30 and 100 days. *T* temperature at two meters (°C), *Td* dew/frost temperature (°C), *Tw* wet-bulb temperature, *Ts* hearth-skin temperature (°C), *H* relative humidity (%), *R* precipitation/rainfall (mm), *Ws* wind speed at two meters (m/s), and *Wd* wind direction at two meters (°)

In this study, the term ‘environment’ refers to one of the 11 farms in the final dataset. The longitude and latitude coordinates were available for each environment, which was used to retrieve daily weather records from the NASA POWER website (https://power.larc.nasa.gov/data-access-viewer/). Taking the measurement day of the trait as ‘day one’, ECs were retrieved for a period of 99 days before the measurement, plus day 1, totaling 100 days of daily weather information for each animal. Mean ECs within 30, 40, 50, 60, 70, 80, 90, and 100 days were considered to account for cumulative weather effects. Since significant regression coefficients (i.e. those different from zero) were found for up to 100 days, further analyses were conducted using 30-day or 100-day daily ECs. The average ECs within each environment for 30 and 100 days are shown in Fig. 1.

**Fig. 2:**
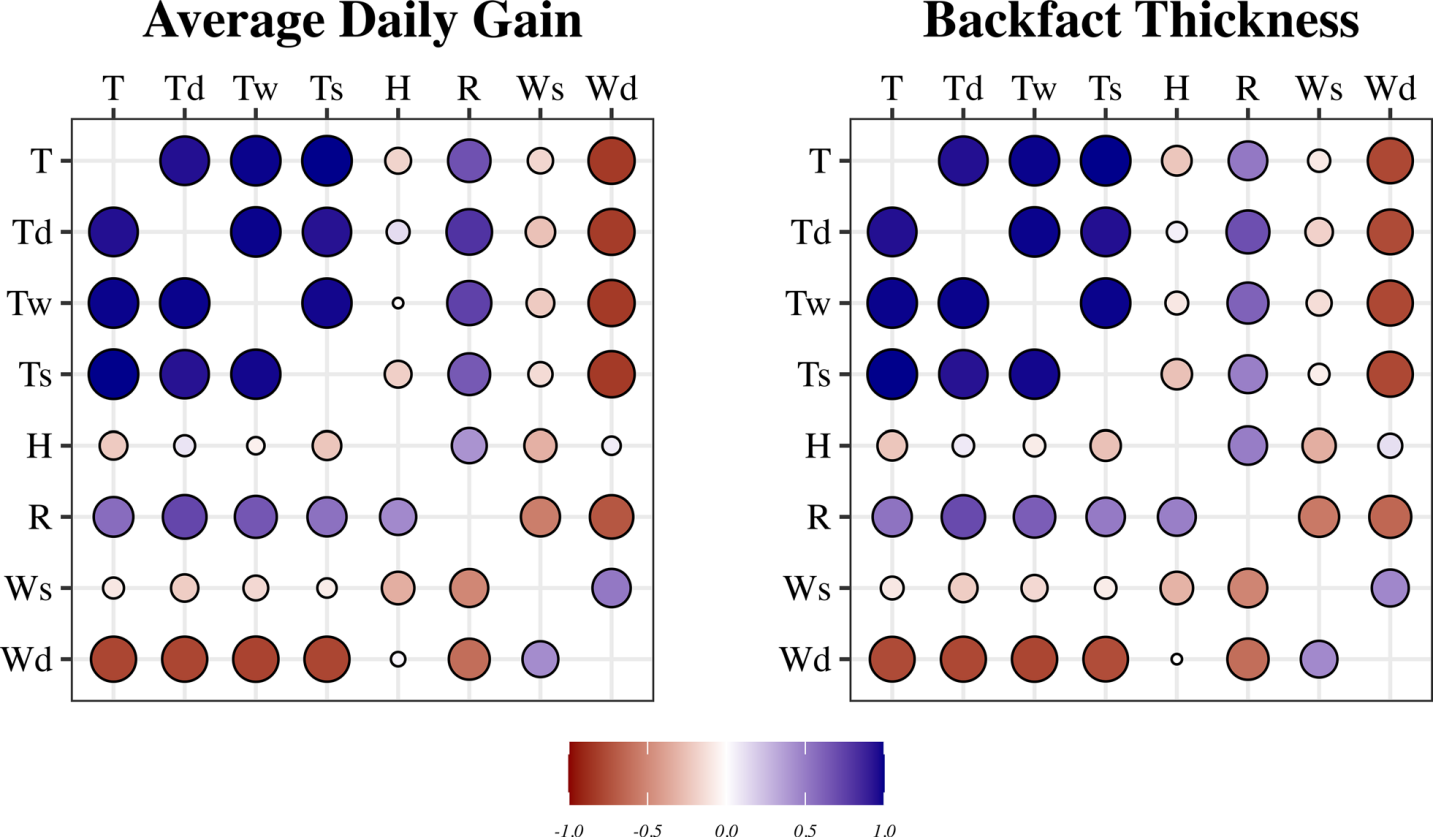
Correlations among environmental covariates (ECs) over 30 days (above diagonal) or 100 days (below diagonal). *T* temperature at two meters (°C), *Td* dew/frost temperature (°C), *Tw* wet-bulb temperature, *Ts* hearth-skin temperature (°C), *H* relative humidity (%), *R* precipitation/rainfall (mm), *Ws* wind speed at two meters (m/s), and *Wd* wind direction at two meters (°)

Of all available ECs, those associated with the phenotype were kept for further analysis. Consequently, eight ECs were used: temperature at 2 m (°C—T), dew/frost temperature (°C—Td), wet-bulb temperature (°C—Tw), earth-skin temperature (°C—Ts), relative humidity (%—H), precipitation/rainfall (mm—R), wind speed at 2 m (m/s—Ws), and wind direction at 2 m (°—Wd). Correlations among ECs for 30 or 100 days are shown in Fig. 2.

### Genomic prediction models

#### Baseline model

Phenotypes (ADG and BFT) were analyzed using the genomic best linear unbiased prediction (GBLUP) mixed model. For both traits, CGs were defined by the concatenation of sex, off-test year, and off-test week. Farm was not included in the CG as it was assumed to capture the environmental effect and was included as a separate random effect. For the two traits, the linear models can be written as:

1 $${\text{ADG}}_{{{\text{ijkl}}}} =\upmu + {\text{CG}}_{{\text{i}}} + {\text{l}}_{{\text{j}}} + {\text{g}}_{{\text{k}}} + {\text{e}}_{{\text{l}}} +\upepsilon _{{{\text{ijkl}}}}$$

2 $${\text{BFT}}_{{{\text{ijkl}}}} =\upmu + {\beta}_{{1}} {\text{W}}_{{\text{k}}} + {\text{CG}}_{{\text{i}}} + {\text{l}}_{{\text{j}}} + {\text{g}}_{{\text{k}}} + {\text{e}}_{{\text{l}}} +\upepsilon _{{{\text{ijkl}}}}$$

where ${\text{ADG}}_{\text{ijkl}}$ or ${\text{BFT}}_{\text{ijkl}}$ denote the phenotypes; $\upmu$ is the intercept; ${\beta}_{1}$ represents the linear regression coefficient of the covariate final weight ($\text{W}$) during the growth test period of the kth animal; ${\text{CG}}_{\text{i}}$ is the fixed effect of the i^th^ contemporary group; ${\text{l}}_{\text{j}}$ is the uncorrelated random effect of the jth litter; ${\text{g}}_{\text{k}}$ is the random additive genetic effect of the kth animal; ${\text{e}}_{\text{l}}$ is the random effect of the lth environment (farm); and ${\upepsilon }_{\text{ijkl}}$ is the random residual term.

In matrix notation, Eqs. 1 and 2 can be written as:

3 $${\mathbf{y}} = {\mathbf{X\boldsymbol{\beta }}} + {\mathbf{Z}}_{1} {\mathbf{l}} + {\mathbf{Z}}_{2} {\mathbf{g}} + {\mathbf{Z}}_{3} {\mathbf{e}} + {{\varvec{\upepsilon}}}$$

where $\mathbf{y}$ represents the vector of phenotypes (ADG or BFT); ${\boldsymbol{\beta }}$ denotes the vector of fixed effects (as defined in Eqs. 1 and 2); $\mathbf{l}$ is the vector of litter effects; $\mathbf{g}$ is the vector of additive genetic effects; $\mathbf{e}$ is the vector of environment effects; ${\varvec{\upepsilon}}$ represents the vector of residual terms; $\mathbf{X}$ and ${\mathbf{Z}}_{\text{i}}$ are the incidence matrices for the fixed and random effects, respectively. Thus, under the assumption of multivariate normality, the random effects (co)variance matrix is given by:

4 $${\text{Var}}\left[ {\begin{array}{*{20}c} {\mathbf{l}} \\ {\mathbf{g}} \\ {\mathbf{e}} \\ {{\varvec{\upepsilon}}} \\ \end{array} } \right] = \left[ {\begin{array}{*{20}c} {{\mathbf{I}}{\upsigma }_{{\text{l}}}^{2} } & {\mathbf{0}} & {\mathbf{0}} & {\mathbf{0}} \\ {\mathbf{0}} & {{\mathbf{G}}{\upsigma }_{{\text{g}}}^{2} } & {\mathbf{0}} & {\mathbf{0}} \\ {\mathbf{0}} & {\mathbf{0}} & {{\mathbf{I}}{\upsigma }_{{\text{e}}}^{2} } & {\mathbf{0}} \\ {\mathbf{0}} & {\mathbf{0}} & {\mathbf{0}} & {{\mathbf{I}}{\upsigma }_{\upepsilon }^{2} } \\ \end{array} } \right]$$

where $\mathbf{I}$ is an identity matrix and $\mathbf{G}$ is the additive genomic relationship matrix, constructed as in VanRaden [32] (method 1). This first model, as presented in Eq. 3 (with assumptions as in Eq. 4), will be referred to as MG (model with uncorrelated environments) in the following.

#### Correlated environments models

The second model incorporates correlated random environmental effects, which can be obtained from the previous model by assuming a (co)variance matrix among environments ($$\mathbf{E}$$) derived from ECs. Let the ECs be stored in a matrix ($$\mathbf{F}$$), with each row corresponding to a different environment and each column containing daily weather measurements ordered by day within covariate. After centering $$\mathbf{F}$$, the product $${\mathbf{FF^{\prime}}}$$ gives the weather (co)variance matrix among environments $$\left( {{\mathbf{E = }}{\mathbf{FF^{\prime}}}} \right)$$; this ensures that $${\overline{\mathbf{E}}}$$$$=0$$. Additionally, $$\mathbf{E}$$ was scaled so $$\overline{{{\text{diag}}\left( {\mathbf{E}} \right)}} = 1$$.

**Fig. 3:**
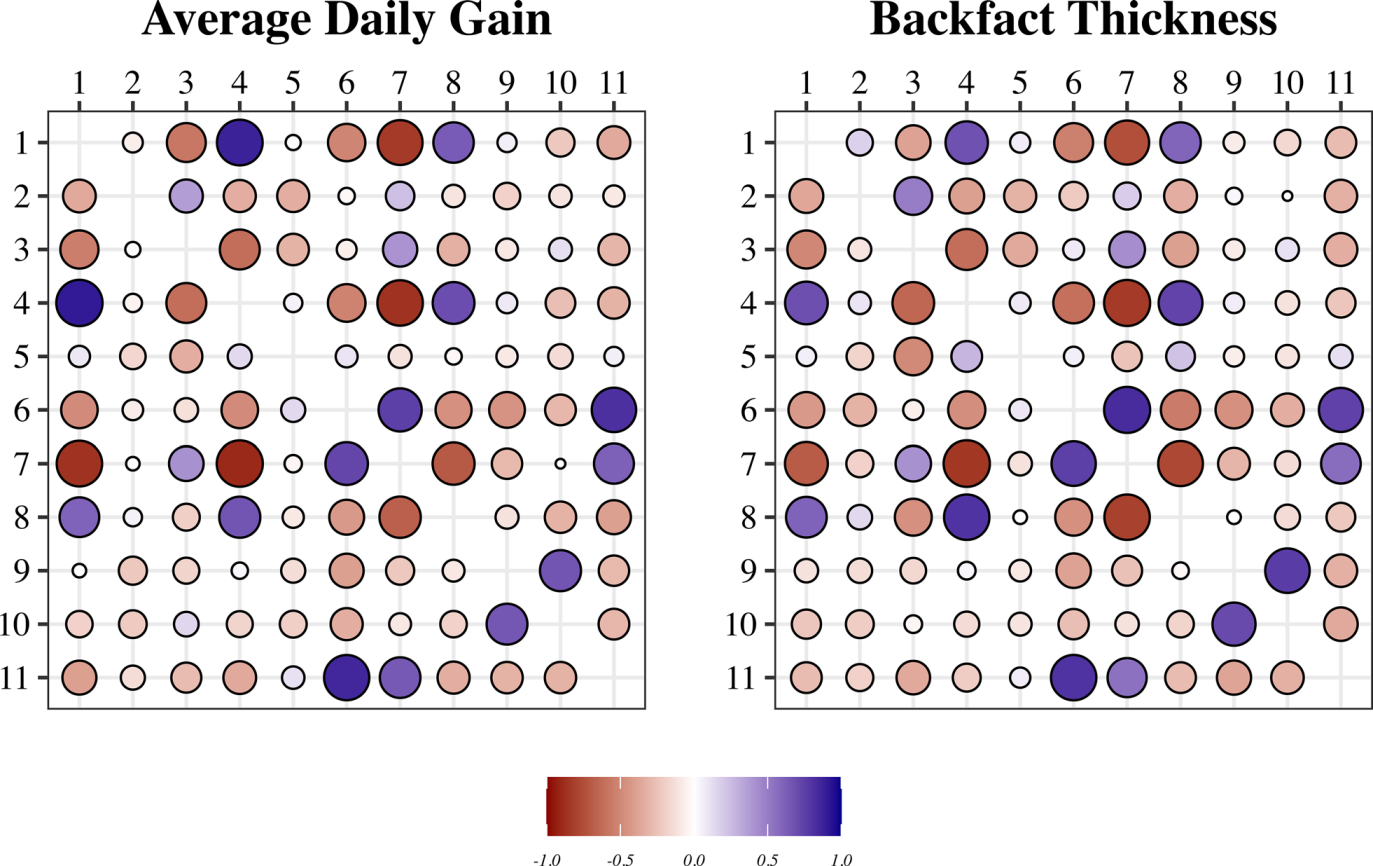
Correlations among environments (farms) based on environmental covariates (ECs) over 30 days (above diagonal) or 100 days (below diagonal)

For this study, two $\mathbf{E}$ matrices were tested based on 30-day weather information (${\mathbf{E}}_{30}$) and 100-day weather information (${\mathbf{E}}_{100}$). Correlations among environments from ${\mathbf{E}}_{\text{i}}$ are presented in Fig. 3. Since the 30-day and 100-day correlations were not similar, we decided to retain both sets for the analysis. These two $\mathbf{E}$ matrices defined two models: ME_30_ (model with correlated environments using 30 days’ worth of ECs), in which $\mathbf{e}\sim \text{MVN}\left(0,{\mathbf{E}}_{30}{\upsigma }_{\text{e}}^{2}\right)$ (MVN: multivariate normal distribution), and ME_100_ (model with correlated environments using 100 days’ worth of ECs), in which $\mathbf{e}\sim \text{MVN}\left(0,{\mathbf{E}}_{100}{\upsigma }_{\text{e}}^{2}\right)$.

#### Genotype-by-environment interaction models

Assuming a first-order multiplicative model, GxE can be modeled by the products of covariance matrices [29]. Using $\mathbf{E}$ as the covariance structure among environments, the environmental (co)variance matrix for animals is given either by ${\mathbf{Z}}_{3} {\mathbf{E}}_{30} {\mathbf{Z}}^{\prime}_{3}$ or by ${\mathbf{Z}}_{3} {\mathbf{E}}_{100} {\mathbf{Z}}^{\prime}_{3} ,$ where ${\mathbf{Z}}_{3}$ is the design matrix defined in Eq. 3 that relates phenotypes to the environments. The GxE can be depicted by the kth additive genetic effect (${\text{g}}_{\text{k}}$) in the lth environment (${\text{e}}_{\text{l}}$), that is ${\text{ge}}_{\text{kl}}$. The $\text{cov}\left({\text{ge}}_{\text{kl}},{\text{ge}}_{{\text{k}}^{\prime}{\text{l}}^{\prime}}\right)$ is equal to $\text{cov}\left({\text{g}}_{\text{k}},{\text{g}}_{{\text{k}}^{\prime}}\right)\text{cov}\left({\text{e}}_{\text{l}},{\text{e}}_{{\text{l}}^{\prime}}\right)$ [29]. Because $\text{Cov}\left(\mathbf{g},\mathbf{g}\right)=\text{Var}\left(\mathbf{g}\right)=\mathbf{G}{\upsigma }_{\text{g}}^{2}$ and ${\text{Cov}}\left( {{\mathbf{Z}}_{3} {\mathbf{e}},{\mathbf{Z}}_{3} {\mathbf{e}}} \right) = {\mathbf{Z}}_{3} {\text{Var}}\left( {\mathbf{e}} \right){\mathbf{Z}}^{\prime}_{3} = {\mathbf{Z}}_{3} {\mathbf{EZ}}^{\prime}_{3} {\upsigma }_{{\text{e}}}^{2} ,$ the covariance structure for the GxE is given by the Hadamard product between $\mathbf{G}$ and ${\mathbf{Z}}_{3} {\mathbf{EZ}}^{\prime}_{3}$. Therefore, including GxE effects, the model was as follows:

5 $${\mathbf{y}} = {\mathbf{X\boldsymbol{\beta} }} + {\mathbf{Z}}_{1} {\mathbf{l}} + {\mathbf{Z}}_{2} {\mathbf{g}} + {\mathbf{Z}}_{3} {\mathbf{e}} + {\mathbf{Z}}_{4} {\mathbf{ge}} + {{\varvec{\upepsilon}}}$$

where $\mathbf{g}\mathbf{e}$ is the vector of GxE random effects. Assuming multivariate normality, the random effects (co)variance matrix is:

6 $${\text{Var}}\left[ {\begin{array}{*{20}c} {\mathbf{l}} \\ {\mathbf{g}} \\ {\mathbf{e}} \\ {{\mathbf{ge}}} \\ {{\varvec{\upepsilon}}} \\ \end{array} } \right] = \left[ {\begin{array}{*{20}c} {{\mathbf{I}}{\upsigma }_{{\text{l}}}^{2} } & {\mathbf{0}} & {\mathbf{0}} & {\mathbf{0}} & {\mathbf{0}} \\ {\mathbf{0}} & {{\mathbf{G}}{\upsigma }_{{\text{g}}}^{2} } & {\mathbf{0}} & {\mathbf{0}} & {\mathbf{0}} \\ {\mathbf{0}} & {\mathbf{0}} & {{\mathbf{E}}_{{\text{i}}} {\upsigma }_{{\text{e}}}^{2} } & {\mathbf{0}} & {\mathbf{0}} \\ {\mathbf{0}} & {\mathbf{0}} & {\mathbf{0}} & {\left( {{\mathbf{G}} \odot {\mathbf{Z}}_{3} {\mathbf{EZ}}^{\prime}_{3} } \right){\upsigma }_{{{\text{ge}}}}^{2} } & {\mathbf{0}} \\ {\mathbf{0}} & {\mathbf{0}} & {\mathbf{0}} & {\mathbf{0}} & {{\mathbf{I}}{\upsigma }_{\upepsilon }^{2} } \\ \end{array} } \right]$$

where ${\upsigma }_{\text{ge}}^{2}$ is the variance component due to GxE effects and $\odot$ denotes the Hadamard product. Two models were tested: MGE_30_ (model considering GxE using 30 days’ worth of ECs), in which $\mathbf{E}={\mathbf{E}}_{30}$, and MGE_100_ (model considering GxE using 100 days’ worth of ECs), where $\mathbf{E}={\mathbf{E}}_{100}$.

#### Multiple-trait model

The multiple-trait model (MTM) models GxE interactions across different environments (farms) using correlated traits. The eleven phenotypes observed on each environment were treated as distinct traits, which enabled estimation of genetic correlations across environments, as well as breeding or genetic values for each animal within each environment. Since the environmental effect is considered by treating phenotypes in the different environments as distinct traits, no environmental effect is explicitly fitted into the model:

7 $$\begin{aligned} \left[ {\begin{array}{*{20}c} {{\mathbf{y}}_{1} } \\ {{\mathbf{y}}_{2} } \\ \vdots \\ {{\mathbf{y}}_{11} } \\ \end{array} } \right] & = \left[ {\begin{array}{*{20}c} {{\mathbf{X}}_{1} } & {\mathbf{0}} & \cdots & {\mathbf{0}} \\ {\mathbf{0}} & {{\mathbf{X}}_{2} } & \cdots & {\mathbf{0}} \\ \vdots & \vdots & \ddots & \vdots \\ {\mathbf{0}} & {\mathbf{0}} & \cdots & {{\mathbf{X}}_{11} } \\ \end{array} } \right]\left[ {\begin{array}{*{20}c} {{{\varvec{\upbeta}}}_{1} } \\ {{{\varvec{\upbeta}}}_{2} } \\ \vdots \\ {{{\varvec{\upbeta}}}_{11} } \\ \end{array} } \right] + \left[ {\begin{array}{*{20}c} {{\mathbf{Z}}_{11} } & {\mathbf{0}} & \cdots & {\mathbf{0}} \\ {\mathbf{0}} & {{\mathbf{Z}}_{12} } & \cdots & {\mathbf{0}} \\ \vdots & \vdots & \ddots & \vdots \\ {\mathbf{0}} & {\mathbf{0}} & \cdots & {{\mathbf{Z}}_{111} } \\ \end{array} } \right]\left[ {\begin{array}{*{20}c} {{\mathbf{l}}_{1} } \\ {{\mathbf{l}}_{2} } \\ \vdots \\ {{\mathbf{l}}_{11} } \\ \end{array} } \right] \\ & + \left[ {\begin{array}{*{20}c} {{\mathbf{Z}}_{21} } & {\mathbf{0}} & \cdots & {\mathbf{0}} \\ {\mathbf{0}} & {{\mathbf{Z}}_{22} } & \cdots & {\mathbf{0}} \\ \vdots & \vdots & \ddots & \vdots \\ {\mathbf{0}} & {\mathbf{0}} & \cdots & {{\mathbf{Z}}_{211} } \\ \end{array} } \right]\left[ {\begin{array}{*{20}c} {{\mathbf{u}}_{1} } \\ {{\mathbf{u}}_{2} } \\ \vdots \\ {{\mathbf{u}}_{11} } \\ \end{array} } \right] + \left[ {\begin{array}{*{20}c} {{{\varvec{\upepsilon}}}_{1} } \\ {{{\varvec{\upepsilon}}}_{2} } \\ \vdots \\ {{{\varvec{\upepsilon}}}_{11} } \\ \end{array} } \right] \\ \end{aligned}$$

where all terms were as previously defined and the subscript i represents the ith environment. Once again, assuming multivariate normality, the (co)variance matrix among random effects is:

8 $${\text{Var}}\left[ {\begin{array}{*{20}c} {\mathbf{l}} \\ {\mathbf{g}} \\ {{\varvec{\upepsilon}}} \\ \end{array} } \right] = \left[ {\begin{array}{*{20}c} {{{\varvec{\Sigma}}}_{0} {\mathbf{ \otimes }}{\mathbf{I}}} & {\mathbf{0}} & {\mathbf{0}} \\ {\mathbf{0}} & {{{\varvec{\Sigma}}}_{1} {\mathbf{ \otimes }}{\mathbf{G}}} & {\mathbf{0}} \\ {\mathbf{0}} & {\mathbf{0}} & {{{\varvec{\Sigma}}}_{2} {\mathbf{ \otimes }}{\mathbf{I}}} \\ \end{array} } \right]$$

where ${{\varvec{\Sigma}}}_{0}$ represents the (co)variance matrix among litter effects across environments (a diagonal matrix in this study); ${{\varvec{\Sigma}}}_{1}$ denotes the additive genetic relationship (co)variance matrix among environments; and ${{\varvec{\Sigma}}}_{2}$ is the residual (co)variance matrix among environments (a diagonal matrix in this study). The GxE was considered negligible, when the estimate of the genetic correlation between environments exceeded 0.80 [24, 25, 33], reflecting that trait expression was similar across different environments.

### Validation

The models were compared using the linear regression (LR) method [34]. Two validation schemes, were implemented: forward validation and environment validation.

#### Forward validation

First, all animals born in 2020 were chosen as focal individuals (i.e. genotyped individuals of interest); therefore, a single ‘whole’ dataset (phenotypes from animals born from 2009 to 2020) and a single ‘partial’ dataset (phenotypes from animals born from 2009 to 2019) were created. The genomic estimated breeding values (GEBV) for the focal group, calculated using the ‘whole’ data were compared with those calculated using the ‘partial’ dataset based on three statistics: accuracy (acc), bias $(\updelta )$, and dispersion $(\text{d}$). These statistics were calculated as follows:

9 $${\text{acc}} = \sqrt {{\text{cov}}\left( {{\hat{\mathbf{g}}}_{{\text{p}}} ,{\hat{\mathbf{g}}}_{{\text{w}}} } \right)/\left( {1 - {\overline{\text{F}}}} \right){\upsigma }_{{\text{g}}}^{2} }$$

10 $${\updelta } = \left( {\overline{{{\hat{\mathbf{g}}}}}_{{\text{p}}} - \overline{{{\hat{\mathbf{g}}}}}_{{\text{w}}} } \right)/{\hat{\sigma }}_{{\text{g}}}$$

11 $${\text{b}_{1}} = {\text{cov}}\left( {{\hat{\mathbf{g}}}_{{\text{p}}} ,{\hat{\mathbf{g}}}_{{\text{w}}} } \right)/{\text{var}}\left( {{\hat{\mathbf{g}}}_{{\text{p}}} } \right)$$

where ${\widehat{\mathbf{g}}}_{\text{p}}$ and ${\widehat{\mathbf{g}}}_{\text{w}}$ represent the vectors of GEBV for the focal individuals from the partial and whole datasets, respectively; $\overline{\text{F} }$ denotes the average pedigree-based inbreeding coefficient for the focal animals, and ${\widehat{\upsigma }}_{\text{g}}$ indicates the estimated genetic standard deviation. For the MTM, the validation was performed separately for each trait and environment and then $\text{acc}$, $\updelta$, and $\text{b}_{1}$ were averaged across environments.

#### Environment validation

Multiple partial datasets were generated for the environment validation by masking phenotypes from each environment, one at the time. Then $\text{acc}$, $\updelta$, and $\text{b}_{1}$ were calculated for each masked environment using the same equations as for forward validation. The same reasoning was applied for the MTM: phenotypes for one environment were masked, GEBV were predicted, and validation statistics were calculated accordingly for the masked environment.

### Software

For all models (MG, ME_30_, ME_100_, MGE_30_, MGE_100_, and MTM), variance components were estimated using restricted maximum likelihood (REML) with genomic information. This process combined the expectation maximization (EM-REML) and average information (AI-REML) algorithms using the BLUPF90 + software [35]. GEBVs were estimated by iteration on data using the BLUP90IOD3 software [35]. For the MTM, variance components were initially estimated using two-trait analyses. Subsequently, the complete (co)variance matrix was obtained by weighted bending using the ITSUMCOV software [36], with weighting factors equal to the number of records in each analysis [37]. The GxE matrices were calculated and inverted using an in-house Fortran program with parallel processing (OpenMP) and Intel Math Kernel Library (MKL).

## Results

### Variance components and heritability

Variance components and heritability estimates are shown in Table 3. Compared to MG, the variance component attributed to the environment (farm) (${\upsigma }_{\text{e}}^{2}$) was greater for ME_30_ and ME_100_. The increase in ${\upsigma }_{\text{e}}^{2}$ was approximately 4.8 times and 5.2 times when comparing MG with all the other models (ME_30_, ME_100_, MGE_30_, and MGE_100_) for ADG and BFT, respectively. For ADG, ${\upsigma }_{\text{e}}^{2}$ represented 14.6% of the phenotypic variance (${\upsigma }_{\text{y}}^{2}$) for MG but increased to 47.8% for ME_30_. This same ratio (${\upsigma }_{\text{e}}^{2}/{\upsigma }_{\text{y}}^{2}$) was much higher for BFT: 43.6% for MG and increasing up to 80.7% for ME_30_. The variance component due to GxE (${\upsigma }_{\text{ge}}^{2}$) accounted for 1.2% and 1.3% of the phenotypic variance in ADG for MGE_30_ and MGE_100_, respectively. For BFT, ${\upsigma }_{\text{ge}}^{2}$ was responsible for 0.5% and 0.6% of the phenotypic variance for MGE_30_ and MGE_100_, respectively. Heritability estimates for ADG ranged from 0.09 (MGE_30_) to 0.16 (MG), while for BFT, the estimates ranged from 0.06 (ME_30_, ME_100_, MGE_30_, and MGE_100_) to 0.18 (MG). Estimates of heritability for ADG and BFT in our subset data were lower and differed somewhat from those reported in the literature, which were based on pedigree-based models [38, 39] or single-step genomic-based models [40].

**Table 3:** Estimates (SE) of variance components, heritability, and environmentability, along with the proportion of phenotypic variance due to GxE for all tested models by trait

Variance component^1^	Model				
	MG	ME_30_	ME_100_	MGE_30_	MGE_100_
ADG					
${\upsigma }_{\text{l}}^{2}$	1967.40 (51.80)	1967.20 (51.80)	1967.20 (51.80)	1901.50 (51.50)	1898.10 (51.46)
${\upsigma }_{\text{g}}^{2}$	1439.10 (72.37)	1439.80 (72.40)	1439.70 (72.40)	1384.70 (74.61)	1385.00 (71.56)
${\upsigma }_{\text{e}}^{2}$	1329.70 (610.22)	7127.16 (3244.33)	5675.50 (2581.81)	7024.17 (3206.59)	5595.28 (2549.52)
${\upsigma }_{\text{ge}}^{2}$				169.91 (26.84)	178.38 (27.14)
${\upsigma }_{\upepsilon }^{2}$	4382.70 (51.80)	4382.60 (40.70)	4382.60 (40.70)	4322.20 (41.23)	4317.40 (41.24)
${\text{h}}^{2}$	0.16 (0.01)	0.10 (0.01)	0.11 (0.01)	0.09 (0.01)	0.10 (0.01)
${\text{e}}^{2}$	0.15 (0.06)	0.48 (0.01)	0.42 (0.03)	0.47 (0.03)	0.42 (0.02)
${\text{ge}}^{2}$				0.01 (0.00)	0.01 (0.00)
BFT					
${\upsigma }_{\text{l}}^{2}$	0.38 (0.01)	0.38 (0.01)	0.38 (0.01)	0.36 (0.01)	0.36 (0.01)
${\upsigma }_{\text{g}}^{2}$	0.84 (0.04)	0.84 (0.04)	0.84 (0.01)	0.79 (0.04)	0.79 (0.04)
${\upsigma }_{\text{e}}^{2}$	2.11 (0.95)	11.38 (5.10)	10.63 (4.80)	11.29 (5.07)	10.53 (4.73)
${\upsigma }_{\text{ge}}^{2}$				0.07 (0.01)	0.08 (0.01)
${\upsigma }_{\upepsilon }^{2}$	1.51 (0.02)	1.51 (0.02)	1.51 (0.02)	1.49 (0.02)	1.48 (0.02)
${\text{h}}^{2}$	0.18 (0.04)	0.06 (0.01)	0.06 (0.01)	0.06 (0.01)	0.06 (0.01)
${\text{e}}^{2}$	0.41 (0.14)	0.81 (0.08)	0.80 (0.09)	0.81 (0.08)	0.80 (0.09)
${\text{ge}}^{2}$				0.01 (0.00)	0.01 (0.00)

*MG* traditional genomic best linear unbiased predictor (GBLUP) model, *ME30* GBLUP considering environmental effects correlated based on 30 days of weather information, *ME100* GBLUP considering environmental effects correlated based on 100 days of weather information, *MGE30* GBLUP considering GxE based on 30 days of weather information, *MGE100* GBLUP considering GxE based on 100 days of weather information, *ADG* average daily gain, *BFT* backfat thickness

^1^ ${\upsigma }_{\text{l}}^{2}$ = variance due to litter; ${\upsigma }_{\text{g}}^{2}$ = additive genetic variance; ${\upsigma }_{\text{e}}^{2}$ = environmental variance; ${\upsigma }_{\text{ge}}^{2}$ = GxE variance; ${\upsigma }_{\upepsilon }^{2}$ = residual variance; ${\text{h}}^{2}$ = heritability; ${\text{e}}^{2}$ = proportion of the phenotypic variance explained by environmental effect-environmentability; ${\text{ge}}^{2}$ = proportion of phenotypic variance explained by the genotype by environmental interaction

### Genetic correlations

Genetic correlations (r_g_) for ADG among environments ranged from 0.03 to 1.00. Conversely, for BFT, these estimates were between 0.35 and 0.83. Among the possible 55 genetic correlation estimates among the 11 environments (based on the MTM), 17 exceeded 0.80 (31%) for ADG (ranging from 0.81 to 1.00), while only four (7%) did so for BFT (ranging from 0.80 and 0.83). Additionally, phenotypes for environments 9, 10, and 11 were weakly genetically correlated (r_g_ < 0.80) with any of the other environments for ADG; in contrast, only environments 2, 4, 7, and 8 were strongly genetically correlated (r_g_ > 0.80) for BFT. Complementary data showing the estimates of the genetic correlations for both traits in the MTM are provided in Additional file 1 (Table S1).

### Validation

#### Forward validation

Except for MTM on BFT, MG, ME_30_, ME_100_, MGE_30_, and MGE_100_ provided similar estimates of $\text{acc}$, $\updelta$, and $\text{b}_{1}$ of GEBV for both traits (Table 4). However, MTM GEBV were 5.5% less accurate for ADG and 27.9% less accurate for BFT. For BFT, ME_100_ had the best estimate of $\text{b}_{1}$ (0.99), while the MTM resulted in underdispersed predictions ($\text{b}_{1}=1.45$). For ADG, MGE_30_, MGE_100_, and MTM exhibited negligible bias compared to the other models ($\updelta$ = 0.01 or 0.02, vs. 0.00). Remarkably, all estimates of $\updelta$ were negative for BFT, with MTM having the smallest estimate (-0.01). Information on model fit is presented in Table 5. Models MGE_100_ and MGE_30_ had a better fit than models MG, ME_30_, and ME_100_ for both traits.

**Table 4:** Validation statistics for all tested models by trait for individuals born in 2020 as focal animals (forward validation)

Validation Statistic	Model					
	MG	ME_30_	ME_100_	MGE_30_	MGE_100_	MTM^1^
ADG						
Accuracy (acc)	0.79	0.80	0.79	0.80	0.79	0.75
Bias (δ)	0.00	0.00	0.00	0.01	0.01	0.02
Dispersion (b_1_)	0.94	0.94	0.94	0.95	0.95	0.97
BFT						
Accuracy (acc)	0.86	0.86	0.86	0.86	0.86	0.62
Bias (δ)	− 0.04	− 0.05	− 0.05	− 0.05	− 0.05	− 0.01
Dispersion (b_1_)	0.97	0.97	0.99	0.97	0.97	1.45

*MG* traditional genomic best linear unbiased predictor (GBLUP) model, *ME*_*30*_ GBLUP considering environmental effects correlated based on 30 days of weather information, *ME*_*100*_ GBLUP considering environmental effects correlated based on 100 days of weather information, *MGE*_*30*_ GBLUP considering GxE based on 30 days of weather information, *MGE*_*100*_ GBLUP considering GxE based on 100 days of weather information, *MTM* multiple trait GBLUP model, *ADG* average daily gain, *BFT* backfat thickness

^1^ Average values across all environments

**Table 5:** Model fit information for all tested models by trait

Model	− 2LogL^1^	AIC^2^	BIC^3^
ADG			
MG	410,039.34	410,047.34	410,081.24
ME_30_	410,044.44	410,052.44	410,086.34
ME_100_	410,043.86	410,051.60	410,085.76
MGE_30_	409,971.06	409,981.06	410,023.43
MGE_100_	409,962.91	409,972.91	410,015.28
BFT			
MG	114,144.04	114,152.04	114,185.52
ME_30_	114,150.35	114,158.35	114,191.83
ME_100_	114,150.70	114,158.70	114,192.18
MGE_30_	114,058.73	114,068.73	114,110.58
MGE_100_	114,048.48	114,058.48	114,100.33

*MG* traditional genomic best linear unbiased predictor (GBLUP) model, *ME*_*30*_ GBLUP considering environmental effects correlated based on 30 days of weather information, *ME*_*100*_ GBLUP considering environmental effects correlated based on 100 days of weather information, *MGE*_*30*_ GBLUP considering GxE based on 30 days of weather information, *MGE*_*100*_ GBLUP considering GE based on 100 days of weather information, *MTM* multiple trait GBLUP model, *ADG* average daily gain, *BFT* backfat thickness

^1^Log likelihood function

^2^Akaike information criteria

^3^Bayesian information criteria

#### Environment validation

**Fig. 4:**
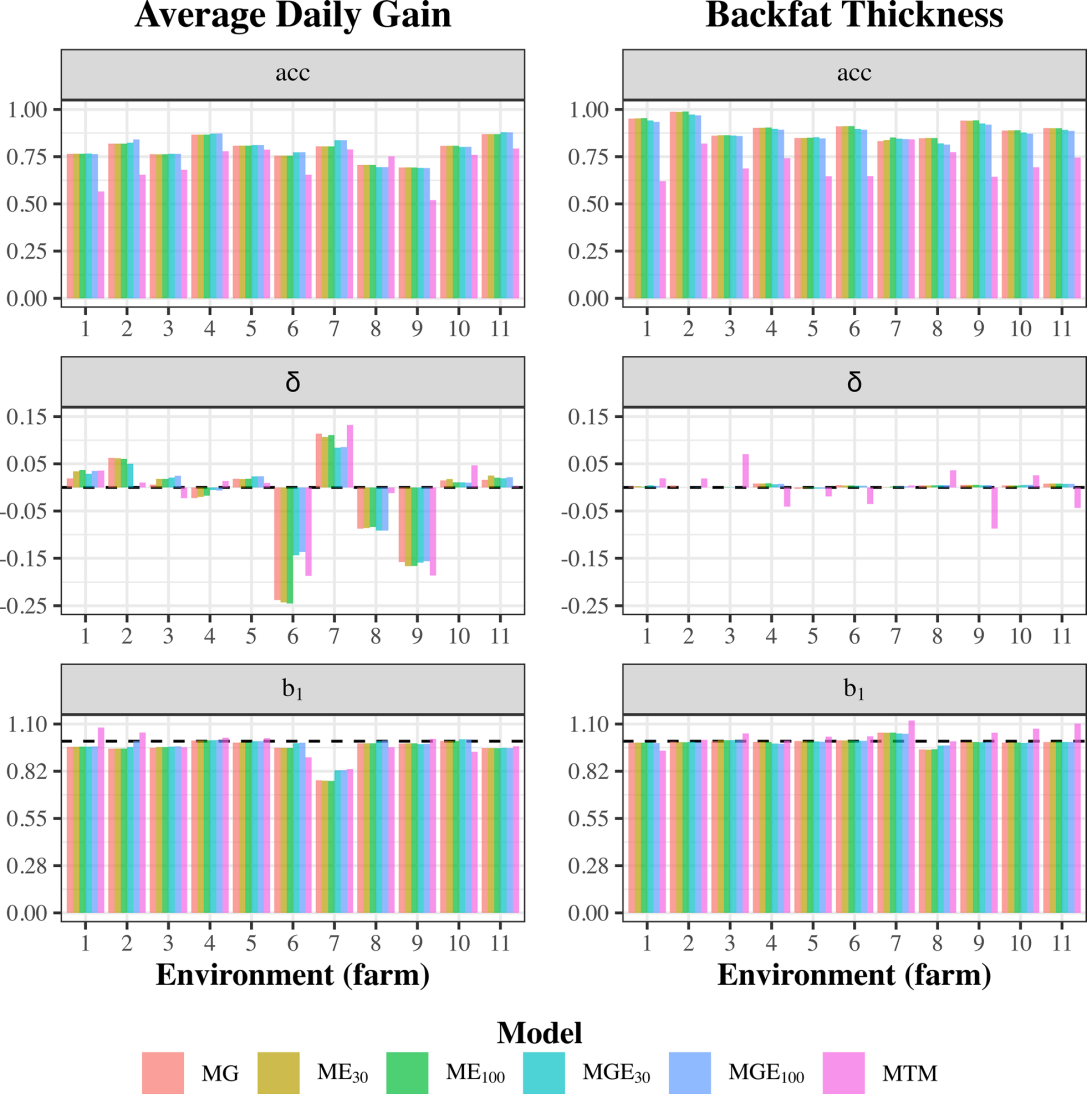
Validation statistics for all tested models by trait, considering all animals in a given environment (farm) as focal individuals (environment-validation). *MG* traditional genomic best linear unbiased predictor (GBLUP) model, *ME*_*30*_ GBLUP considering environmental effects correlated based on 30 days of weather information, *ME*_*100*_ GBLUP considering environmental effects correlated based on 100 days of weather information, *MGE*_*30*_ GBLUP considering genotype by environment interaction (GxE) based on 30 days of weather information, *MGE*_*100*_ GBLUP considering GxE based on 100 days of weather information, *MTM* multiple-trait GBLUP model, *ADG* average daily gain, *BFT* backfat thickness

For ADG, the models MG, ME_30_, ME_100_, MGE_30_, and MGE_100_ showed similar estimates of acc for all environments, except for environment 7 (Fig. 4). For BFT, MGE_30_ and MGE_100_ exhibited marginally lower acc across all environments. The MTM had the smallest acc of GEBV in all environments for both traits, except for ADG in environment 8. Bias was negligible for BFT for all models, while environments 6, 7, 8, and 9 displayed the highest bias levels for ADG. Additionally, the estimate of $\text{b}_{1}$ was close to 1.00 for BFT for all environments, except for environment 7. For BFT, GEBV from the MTM were either under-dispersed (environments 3, 5, 6, 7, 9, 10, and 11) or overdispersed (environments 1 and 8). Predictions for ADG were generally overdispersed across models and environments, except for environments 1, 2, 4, and 5 under the MTM.

## Discussion

This study aimed to evaluate the validity of high-dimensional environmental data for modeling correlated environments and GxE related to production traits in pigs. The results indicated that incorporating ECs into the genomic prediction model did not significantly enhance the prediction accuracy of GEBV for ADG and BFT, despite an improvement in model fit. In this study, we used external ECs while the animals were kept indoors, which may have limited the benefits of using these ECs. Our findings suggest that, although incorporating ECs into the prediction model improves its overall fit, the advantage for genomic predictions, specifically prediction accuracy, remains limited. However, we found some increase in prediction accuracy for BFT when comparing ME and MGE versus MTM, suggesting that the benefit of using ECs might be trait-dependent, thus highlighting the need for future research with in-barn ECs and other traits.

Purebred animals are typically raised in better environments than commercial pigs. However, environmental differences can exist even when building conditions (e.g. cooling and humidity) and animal conditions (e.g. density) are controlled. Previous studies have shown that even purebred pigs are susceptible to heat stress [2] and show some degree of GxE [17]. We also found significant effects of external weather conditions, although we used only purebred animals from well-controlled nucleus herds in this study. Therefore, we speculate that external ECs can interfere with the microclimate within barns, which may indirectly affect the animals’ performance.

### Environmental covariates

Using THI as the sole EC, Fragomeni et al. showed that 30 days was sufficient to model the response to heat stress [2] and, subsequently, the GxE [17]. In recent studies on dairy cattle, McWhorter et al. and Campos et al. and averaged THI over 5 and 3 days, respectively, as an environmental descriptor [18, 19]. The THI is strongly correlated with temperatures [21, 22] and all ECs were correlated (to some degree) in this study (Fig. 2). Therefore, we expected that regressing the phenotype on the average of an EC would not result in significant results in the last 30 days before the phenotype is collected. However, we found an association between the phenotype and the ECs for up to 100 days. This outcome indicates that the environmental impact on the phenotype is not solely a result of immediate climate conditions but is also influenced by continuous exposure.

Campos et al. argued that wind speed and solar radiation might influence heat stress conditions experienced by animals, especially when kept in outdoor conditions [19]. They also suggested that in-barn conditions could limit this influence. All temperatures in this study (T, Td, Tw, and Ts) showed a weak negative correlation with Ws (Fig. 2). Conversely, a strong negative correlation with Wd was found (Fig. 2). Ws and Wd exhibited a moderate positive correlation (Fig. 2), indicating that the temperature can decrease rapidly when the external temperature is high, depending on wind direction and speed. This observation helps clarify the positive regression coefficient of the phenotype on Ws and Wd. Furthermore, it explains why all EC related to temperature had less impact than Ws.

Given the correlations among H and all ECs related to temperature (Fig. 2), one could expect little to no influence from the increased humidity when temperature ECs are elevated. The correlations among R and all temperature ECs (Fig. 2) indicate that an increase in temperature generally leads to more rain, resulting in a rise in H (moderate positive correlation between R and H), potentially triggering heat-stress responses. The THI effectively captures this behavior by combining increased H (due to more R) with rising temperatures. By not using the ECs through an index calculation, the dynamic nature of climate conditions was incorporated into the herd effect and the GxE term. However, fitting multiple ECs and modeling a random regression coefficient for each would increase the complexity of the model, the number of parameters, and the computing cost, which explains the popularity of the THI as a singular environmental gradient in previous studies [17, 18, 20]: rather than fitting one regression for each EC, only one is needed. Moreover, by modeling only the linear random regression coefficient of the HL, there is no need to use orthogonal polynomials or to standardize the covariates [41], which are needed when using several ECs.

Considering each EC for each day as a different covariate through the traditional reaction-norm approach would necessitate at least 240 (eight ECs over 30 days) or 800 (eight ECs over 100 days) random regression coefficients, especially as we used daily ECs to construct **E**. By modeling the ECs using covariance functions [29], the number of parameters was significantly reduced, resulting in an equivalent model as a standard reaction norm that fits one linear random regression coefficient for each EC. Incorporating daily ECs into the calculation of **E** also allowed us to account for the temporal variability within either 30- or 100-day periods. In preliminary analyses (data not shown), we observed that using over 100 days of daily ECs yielded the same variance components as those presented in Table 3 and resulted in similar validation statistics for GEBV (Table 4).

Jarquín et al. accounted for temporal environmental effects by defining each environment as a combination of location and year [29]. In contrast, our approach integrated the temporal effect with the environmental gradient on a daily basis. In Jarquín et al.’s approach, within-year differences, including seasonal or daily variations, are not accounted for, whereas our approach averages differences across years within each location. Additionally, the yearly effect on phenotype was included in the CG, which could have introduced confounding between CG and the random environment effect if we had defined the environment as farm concatenated with year. Although we do not anticipate significant annual environmental variations to affect the studied traits and given that the growth trial duration influences the phenotype, the potential to account for both within- and across-year variation warrants further investigation.

### Variance components and heritability

When not fitting the random environmental effect in the model (data not shown), heritability estimates were 0.19 for ADG and 0.30 for BFT. Previous works with different populations of purebred and crossbred pigs reported heritability estimates for ADG varying from 0.15 to 0.30 and for BFT from 0.41 to 0.56 [38, 39]. It is worth noting that differences in heritability estimates could arise because of data structure and editing, as well as from the model used. We used single-trait genomic models in our study, while Steyn et al. and Leite et al. used multiple-trait pedigree-based models [38, 39]. Using or omitting genomic information when estimating variance components can lead to biased estimates, particularly if the population is undergoing selection [42–44]. It is, therefore, not unrealistic to assume such factors could result in the lower heritability estimates found in our study, along with the fact that our data represents a subset of the population.

When environments were included in the model as uncorrelated random effects (MG), there was a reduction in the variance components attributable to litter (${\upsigma }_{\text{l}}^{2}$) and additive genetic (${\upsigma }_{\text{g}}^{2}$) effects (data not shown). There was also an increase in phenotypic variance, while the residual variance remained unchanged compared with a model without herd effects. Thus, this reduced heritability estimates (0.16 for ADG and 0.17 for BFT). This indicates potential confounding between herd and litter effects [45]. Animals typically do not have records in multiple environments unless there is a temporal change and litters also do not exist in multiple herds simultaneously. Hence, the presence of animals and litters within a single herd could explain the reduction in ${\upsigma }_{\text{l}}^{2}$ and ${\upsigma }_{\text{g}}^{2}$ when environment was included in the model as a random effect.

Confounding between effects that are included in a model makes it difficult to obtain separate estimates for them. Gelfand and Sahu noted that Bayesian non-identifiability is equivalent to the lack of identifiability in likelihood methods [46]. For a model with a parameter vector $${\mathbf{\theta^{\prime}}} = \left[ {\begin{array}{*{20}c} {{\uptheta }_{1} } & {{\uptheta }_{2} } \\ \end{array} } \right]^{\prime} ,$$ Bayesian non-identifiability means that what can be inferred about ${\uptheta }_{2}$ depends on ${\uptheta }_{1}$ [47]. Identifiability conditions in likelihood-based approaches are well studied [48–52]. For a variance component vector $${\mathbf{\theta^{\prime}}} = \left[ {\begin{array}{*{20}c} {{\uptheta }_{1} } & {{\uptheta }_{2} } & \cdots & {{\uptheta }_{{\text{n}}} } \\ \end{array} } \right]^{\prime}$$ and associated (co)variance matrices ${\mathbf{V}}_{1},{\mathbf{V}}_{2},\cdots ,{\mathbf{V}}_{\text{n}}$, identifiability requires a positive and definite restricted maximum likelihood (REML) information matrix, which implies that the matrices **V** need to be linearly independent [49, 50]. In our models, the average information REML (AI-REML) information matrix was positive-definite, indicating that the models were identifiable. However, the environment (co)variance matrix showed some dependence on the litter (co)variance matrix due to litter nested within the environments. This issue could be resolved by observing the same animal across environments (e.g. through cloning, which is often not feasible) or by removing litter effects. However, the latter will introduce bias.

Using $\text{Var}\left(\mathbf{e}\right)=\mathbf{E}{\upsigma }_{\text{e}}^{2}$ rather than $\text{Var}\left(\mathbf{e}\right)=\mathbf{I}{\upsigma }_{\text{e}}^{2}$ resolved identifiability issues by allowing environment-specific variation. Notably, the environment (co)variance matrix rank improved when environmental correlations were considered (MG: rank 11; ME_30_: rank 15; and ME_100_: rank 20). However, in the ME_30_ model, environments were completely independent of litter, resulting in the smallest condition number of the AI-REML information matrix. This aligns with the suggestion that 30 days of weather data most effectively captures GxE and heat-stress effects in pigs [2, 17]. This also suggests fitting ECs in the genomic prediction model may provide theoretical modeling improvements. However, its predictive benefit may be limited.

The lower heritability estimates for ME and MGE compared to MG was similar to that reported by Makanjuola et al. [1] and Cuyabano et al. [13]. Furthermore, Makanjuola et al. found comparable results for the same traits in the same population but instead used the THI to correlate herd effects [1]. Conversely, Tiezzi et al. did not find increases in ${\upsigma }_{\text{e}}^{2}$ when comparing a model that included only environmental effects to one that included both environmental and genetic effects [14]. Tiezzi et al. [14] used a sire model, while Makanjuola et al. [1] and Cuyabano et al. [13] used an animal model. Under a sire model, records on different daughters act as repeated records for their sire. This helps mitigate potential partial confounding between random effects, as sires typically have progeny across multiple herds.

If the number of observations per level of a given environmental effect is sufficiently large, modeling it as fixed or random does not significantly impact prediction accuracy of EBV [53]. However, when the number of records per subclass is limited, modeling it as random is preferred, as a reduction in prediction error variance is expected [54, 55]. Moreover, when modeling an effect such as this as a fixed effect, its variance is not included in the heritability calculation; therefore, when treated as random, its variance should also be omitted in the heritability estimation. When taking this into account, heritability estimates in our study remained stable across all models (data not shown), regardless of whether ${\upsigma }_{\text{e}}^{2}$ increased or not.

Pigs face not only climatic factors, but also pathogen load, management practices, and interactions with other animals are also part of the environmental effect. It may be that the assumption that all environment variance is accounted for in the $\mathbf{E}$ matrix is too strong, causing ${\upsigma }_{\text{e}}^{2}$ to increase. To understand this, models ME_30_, ME_100_, MGE_30_, and MGE_100_ were re-run with an extra uncorrelated random effect for the environment (ME_30_*, ME_100_*, MGE_30_*, and MGE_100_*) (Additional file 2, Table S2, and Additional file 3, Table S3). Thus, the environment was split into two parts: one modeled by the climate (ECs) and one uncorrelated part. This did not increase the phenotypic variance (i.e. ${\upsigma }_{\text{y}}^{2}$) hence, the estimate of heritability was the same for all models. When we examined the model fit (Table 5 and Table S3), the benefit of including this non-climate (or microenvironment) effect was confirmed. Jarquín et al. suggested this approach to account fully for the environmental effect on grain yield data [29]. Validation statistics (accuracy, bias, and dispersion) did not change for these models (ME_30_*, ME_100_*, MGE_30_*, and MGE_100_*) in our data.

Initially, modeling correlated environmental effects through ECs was proposed in the context of plant breeding [29] and it has been successfully applied in several crops under selection, using pedigree- and genomic-based prediction [56–61]. These studies included repeated records both within and across environments. In plants, the ability to propagate through sexual and asexual reproduction (i.e. vegetative propagation and cloning) facilitates testing the same genotype across several environments. Using (co)variance structures for the environmental effect enables prediction of an individual’s performance in environments where their phenotype was not observed [58]. However, this prediction has low accuracy if no individual is measured in multiple environments, as was the case for our data.

### Genetic correlations and genotype-by-environment interaction

For ADG, 31% of the genetic correlation estimates among environments exceeded 0.80, whereas only 7% did so for BFT. Although the MTM was feasible in our study, when the number of environments is too large, treating each environment as a different trait is impossible because (1) the genetic (co)variance matrix can become non-positive-definite and (2) it requires more computing power. Therefore, our approach using ECs is preferable in such cases as it addresses correlated environmental effects and GxE by fitting additional random effects without significantly increasing computing costs. Fitting an MTM also requires that each environment has sufficient records, which is not always achievable. Record insufficiency can decrease the prediction accuracy in MTM and bias estimates of variance components.

When the number of records for each environment (i.e. farm) is limited, estimates of variance components have a high standard error. If some covariances are poorly estimated, the animals from those environments do not fully contribute to the accuracy of the animals in the other environments, leading to lower accuracy when using the MTM. One way to address this issue is by removing farms with too few records (e.g., fewer than 2000). However, doing so would reduce the total number of records and the number of environments for the MTM, making comparisons with other models unfair, as those models would have more records per farm and more farms overall. Moreover, the additive genetic (co)variance matrix used in the MTM had to be bend to ensure it was positive-definite. This adjustment could disrupt the covariance estimates when the number of records per trait (or environment, in this case) is smaller [36, 37]. In addition, the estimates of genetic correlations between environments in the MTM may not accurately reflect the true genetic correlations between environments. Utilizing pedigree information would allow for the full dataset that includes phenotyped animals that are not genotyped to be analyzed for variance component estimation.

In the present study, the ranking of the animals was consistent across all environments for ADG, indicating that although GxE was identified (see log-likelihood in Table 5), selection and gain were not compromised when ignoring these interactions. However, small changes in the ranking were observed for BFT, suggesting that the type of GxE for BFT differs from that of the ADG, potentially due to management, feeding, and measurement systems affecting BFT more than ADG. Robertson stated that ‘no interaction means a genetic correlation of unity’, but considering its standard error, a correlation smaller than 0.80 would hold biological significance [33]. In later studies, both Mulder and Bijma, and Mulder et al. found no improvement in genetic gain when considering two environments individually if their genetic correlation was greater than 0.80 [24, 25]. These findings may suggest that evaluating the environments individually could maximize the gain; however, the amount of re-ranking across environments in our study for BFT was small and thus did not justify selection within a specific environment. Although, the ranking of animals based on GEBV may remain identical within a trait between environments, the scale of the GEBV might influence the relative contribution of that single trait on the index.

GxE can be defined as the differential expression of genotypes across environments [62], indicating that the phenotype for the same genotype can vary depending on the environment. With presence of GxE, the genetic correlation for a given trait between the environments will deviate from unity. Factors such as location, management, housing, time, and climate can be used to account for the environmental effect [17, 18, 62]. Furthermore, if there is a continuous factor (e.g. time, weather conditions, THI), a random regression model can be used to estimate a slope of the GEBV over the environmental gradient [18]. If there is no gradient (i.e. the environments are categorical), an MTM can be employed to include GxE in the evaluation [27]. However, changes in the GEBV resulting from the environmental effect depend on the type of interaction and may vary according to the diversity of genotypic and environmental effects [62].

Haldane specified several different forms that GxE might take [63]. Lerner grouped Haldane’s classes of interaction into two distinct groups [64]: linear interactions, where genetic and environmental effects are additive, and non-linear otherwise. If the change in genotype from one environment to another is consistent across all genotypes, there will be no re-ranking, and the genetic correlations among environments may remain high. However, the genetic correlations will be less than one when changes differ among genotypes, regardless of whether re-ranking occurs.

Jarquín et al. demonstrated that modeling the environmental effects and GxE by (co)variance functions is equivalent to a reaction norm [29]. In a reaction-norm model, the GEBV is predicted along with the random gradient [17, 18], which, in the context of this study, corresponds to weather information. Following this reasoning, the ‘ge’ breeding value can be interpreted as the amount of change in the GEBV attributed to the environmental effect. Since this value is specific to each environment, it can provide targeted insights for selection decisions. For example, it can guide breeders when sending boars or semen to another herd. Fragomeni et al. applied reaction norms to their investigation into GxE regarding heat stress in pigs [17]. Their study reported, for purebred data, that reaction norms were not effective in demonstrating evidence of GxE since there is rigorous environmental control in nucleus farms. The ECs used in this study reflect outdoor conditions, while the animals were kept indoors. It is possible that, although the MTM showed some GxE, the (co)variances approach as a reaction norm yields better results if the ECs were obtained inside the barn.

The main limitation of the models we used in this study is related to the mode of interaction, in that ‘only multiplicative reaction norm model’ was considered [29]. This model becomes an approximation in the presence of different interaction forms between genetic marker effects and ECs. Correlating herds in an animal breeding context also approximates the uncertain relationship between herds [13]. While modeling environments as correlated random effects accounted for a significant portion of phenotypic variance without negatively impacting breeding value prediction accuracy, it was still not descriptive enough to increase prediction accuracy. It is worth noting that while there was a reduction in heritability due to an increase in the environmental variance (ME_30_, ME_100_, MGE_30_, and MGE_100_), our study did not show a reduction in additive genetic variance and/or prediction accuracy. This outcome may be attributed to the absence of re-ranking of GEBV across environments. If GxE is strong and re-ranking occurs, ignoring these interactions and the potential relationship among herds can result in heritability overestimation and inflated values of expected genetic gain [1, 13].

### Validation

Modeling environments as correlated random effects can increase predictivity [1, 13, 14], defined as the correlation between adjusted phenotypes from a full dataset and GEBV from a partial dataset. This approach relies on model fit and the quality of estimation of fixed effects, reflecting the model’s ability to predict phenotypes [34]. Conversely, the LR method tests the impact of recent data on breeding value predictions [65] and remains robust even with mis-specified effects or heritability [66]. In forward validation, our accuracies surpassed those of Steyn et al. [38] and Leite et al. [39] and aligned with those of Jang et al. [67, 68]. No accuracy improvements were observed across MG, ME, and MGE, indicating that adding weather information to model GxE has minimal benefit for breeding value prediction for ADG and BFT in pigs.

However, for BFT, alternative models outperformed the MTM. This finding suggests that treating phenotype across these environments as a single trait with an environmental effect improves accuracy for traits that have low genetic correlations across environments. Regarding environment validation, the models MG, ME, and MGE outperformed MTM, exhibiting higher accuracy, lower bias, and less dispersion issues. The MTM was constrained by data scarcity in certain environments, which hindered validation performance, particularly those with fewer records. Moreover, bias, accuracy, and dispersion depend on data availability, helping to explain MTM’s lower performance. Modeling phenotype across different environments as a single trait increases the information available, which is especially beneficial for smaller environments. However, in the case of MTMs and when genetic correlations between environments are low, there is no information sharing, leading to reduced prediction accuracy in environments with limited data.

### Practical remarks

The occurrence of GxE observed in plants and animals, although its extent and nature vary. Plants can exhibit a high degree of phenotypic plasticity, adjusting their development based on environmental cues. For example, a plant may exhibit different growth forms, leaf shapes, or flowering times depending on factors like light, temperature, and soil conditions. Conversely, animals generally have a more limited ability to adapt to different environments, with many animal traits strongly influenced by intrinsic genetic factors; therefore, environmental effects may play a smaller role.

In addition, livestock animals are often more mobile than plants and their environment is controlled to some degree; thus, they may not be as directly exposed to or influenced by their immediate environment during trait development. Nevertheless, animals can exhibit some degree of GxE that may or may not change the ranking of GEBV from one environment to another. However, it is crucial to note that GxE is a complex, multifaceted phenomenon that varies among species, populations, and traits. Therefore, it would be inaccurate to claim that GxE is entirely absent in animals or to ignore its impacts; rather, it is suggested that the degree, nature, and extent of these interactions can widely vary.

Currently, most genetic predictions for purebred pigs partially consider differences in the production environments (e.g. management, climate, and social aspects of production) by fitting the farm in the fixed CG. Moreover, GxE interactions are considered negligible for production traits such as ADG and BFT. However, if GxE interactions are present but ignored, prediction accuracy may be compromised, along with over or under-dispersed predictions for young individuals.

Our approach considered high-dimensional environmental data to account for correlations among herds and GxE. This high dimensionality arose from using eight different ECs, combined with daily information. This methodology allowed us to estimate an ‘overall’ breeding value and an ‘environmental specific’ breeding value. The environmental specific EBV should be interpreted as the change in the overall breeding value attributable to environmental effects. If a given genotype has records in each environment, this approach can potentially increase prediction accuracy. We foresee that this model will perform better when there are repeated records, but this remains to be evaluated. MGE_i_ models (or any model that considers GxE) predicts one environmental breeding value for each environment for each animal. We recommend utilizing these environmental-specific EBV for mating and culling decisions while reserving the overall breeding value for selection decisions. From a practical perspective, once new phenotypes and/or environments are added to the data, the environmental covariance structure should be updated, leading to a refresh of all model configurations.

## Conclusions

Modeling environments (herds) as a correlated random effect does not provide any benefit for genomic predictions in pigs. Furthermore, the computing requirements also increase as the model becomes more complex. For traits in which GxE is stronger, modeling GxE by covariance functions is more effective than using the MTM due to the amount of information each environment contributes to the data. Although there may be no immediate practical benefit, models that incorporate GxE can improve mating decisions by predicting how the future offspring of a particular boar or sow perform in different environments. Provided there are records for the same genotype across all environments, the first-order multiplicative (co)variances approach used in this study is expected to yield higher accuracies of EBV than traditional models when there is GxE. Moreover, the traditional MTM in the presence of GxE requires larger datasets and better genetic connections among environments to provide accurate breeding value predictions. Lastly, incorporating environments (herd) into the model as an uncorrelated random effect is sufficient to account for environmental effects and GxE in pigs.

## Supplementary Information

**Additional file 1: Table S1.** Heritability estimates (diagonal, bold) and genetic correlations among all environments (after bending) according to the studied trait. Results contain the bent correlation matrix among all eleven environments for each trait (ADG or BFT) for the multiple trait model.

**Additional file 2: Table S2.** Variance components (SE), heritability (SE), and environmentability (SE) estimates, along with the proportion of phenotypic variance due to GxE for all tested models, including an extra uncorrelated environmental random effect for ME_30_, ME_100_, MGE_30_, and MGE_100_ according to the studied trait. Description: Results contain estimated variance components for all models when an extra uncorrelated random environmental effect was included for ME_30_, ME_100_, MGE_30_, and MGE_100_.

**Additional file 3: Table S3.** Model fit information for all tested models, including an extra uncorrelated environmental random effect for ME_30_, ME_100_, MGE_30_, and MGE_100_ according to the studied trait. Results contain estimated variance components for all models when an extra uncorrelated random environmental effect was included for ME_30_, ME_100_, MGE_30_, and MGE_100_.

## Data Availability

The used datasets are the property of the Pig Improvement Company and are not available publicly.
