# Supplementary material for: Genotype-by-environment interaction with high-dimensional environmental data: an example in pigs
Source: Genet Sel Evol. 2025 Jun 5;57:28. doi: 10.1186/s12711-025-00974-2 (PMC12142960; doi:10.1186/s12711-025-00974-2)
Supplement: Supplementary file 1 — Additional file 1: Table S1. Heritability estimates (diagonal, bold) and genetic correlations among all environments (after bending) according to the studied trait. Results contain the bent correlation matrix among all eleven environments for each trait (ADG or BFT) for the multiple trait model. [file 12711_2025_974_MOESM1_ESM.docx]

**Table S1 Heritability estimates (diagonal, bold) and genetic correlations among all environments (after bending) according to the studied trait**

| E^1^ | E^1^ | | | | | | | | | | |
| --- | --- | --- | --- | --- | --- | --- | --- | --- | --- | --- | --- |
|  | 1 | 2 | 3 | 4 | 5 | 6 | 7 | 8 | 9 | 10 | 11 |
| ADG | | | | | | | | | | | |
| 1 | **0.09** | 0.86 | 0.47 | 0.68 | 0.66 | 0.13 | 0.52 | 0.48 | 0.20 | 0.52 | 0.03 |
| 2 |  | **0.27** | 0.70 | 0.76 | 0.92 | 0.51 | 0.75 | 0.72 | 0.26 | 0.65 | 0.37 |
| 3 |  |  | **0.22** | 0.90 | 0.83 | 0.68 | 1.00 | 0.84 | 0.56 | 0.78 | 0.76 |
| 4 |  |  |  | **0.24** | 0.77 | 0.60 | 0.92 | 0.85 | 0.64 | 0.85 | 0.67 |
| 5 |  |  |  |  | **0.26** | 0.72 | 0.87 | 0.85 | 0.32 | 0.74 | 0.60 |
| 6 |  |  |  |  |  | **0.22** | 0.69 | 0.87 | 0.52 | 0.75 | 0.84 |
| 7 |  |  |  |  |  |  | **0.19** | 0.87 | 0.56 | 0.81 | 0.75 |
| 8 |  |  |  |  |  |  |  | **0.16** | 0.67 | 0.96 | 0.85 |
| 9 |  |  |  |  |  |  |  |  | **0.30** | 0.78 | 0.62 |
| 10 |  |  |  |  |  |  |  |  |  | **0.25** | 0.76 |
| 11 |  |  |  |  |  |  |  |  |  |  | **0.20** |
| BFT | | | | | | | | | | | |
| 1 | **0.36** | 0.65 | 0.64 | 0.65 | 0.35 | 0.54 | 0.67 | 0.69 | 0.63 | 0.63 | 0.65 |
| 2 |  | **0.04** | 0.72 | 0.76 | 0.76 | 0.71 | 0.78 | 0.80 | 0.71 | 0.71 | 0.74 |
| 3 |  |  | **0.33** | 0.76 | 0.75 | 0.70 | 0.78 | 0.79 | 0.70 | 0.70 | 0.73 |
| 4 |  |  |  | **0.47** | 0.77 | 0.72 | 0.81 | 0.82 | 0.74 | 0.72 | 0.77 |
| 5 |  |  |  |  | **0.07** | 0.54 | 0.79 | 0.76 | 0.74 | 0.74 | 0.76 |
| 6 |  |  |  |  |  | **0.53** | 0.73 | 0.74 | 0.67 | 0.68 | 0.69 |
| 7 |  |  |  |  |  |  | **0.46** | 0.83 | 0.76 | 0.76 | 0.79 |
| 8 |  |  |  |  |  |  |  | **0.23** | 0.76 | 0.75 | 0.79 |
| 9 |  |  |  |  |  |  |  |  | **0.36** | 0.67 | 0.70 |
| 10 |  |  |  |  |  |  |  |  |  | **0.20** | 0.70 |
| 11 |  |  |  |  |  |  |  |  |  |  | **0.42** |

^1^Environment. Values highlighted in blue show genetic correlation greater than 0.80.
