# Supplementary material for: Genotype-by-environment interaction with high-dimensional environmental data: an example in pigs
Source: Genet Sel Evol. 2025 Jun 5;57:28. doi: 10.1186/s12711-025-00974-2 (PMC12142960; doi:10.1186/s12711-025-00974-2)
Supplement: Supplementary file 2 — Additional file 2: Table S2. Variance components (SE), heritability (SE), and environmentability (SE) estimates, along with the proportion of phenotypic variance due to GxE for all tested models, including an extra uncorrelated environmental random effect for ME30, ME100, MGE30, and MGE100 according to the studied trait. Description: Results contain estimated variance components for all models when an extra uncorrelated random environmental effect was included for ME30, ME100, MGE30, and MGE100. [file 12711_2025_974_MOESM2_ESM.docx]

**Table S2 Variance components (SE), heritability (SE), and environmentability (SE) estimates, along with proportion of phenotypic variance due to genotype-by-environment interaction for all tested models including an extra uncorrelated environmental random effect for ME_30_, ME_100_, MGE_30_, and MGE_100_ according to the studied trait**

| **Variance Components^1^** | **Models** | | | | |
| --- | --- | --- | --- | --- | --- |
|  | **MG** | **ME_30_** | **ME_100_** | **MGE_30_** | **MGE_100_** |
| ADG | | | | | |
| $\sigma_{l}^{2}$ | 1967.40  (51.80) | 1967.30  (51.80) | 1967.30  (51.80) | 1901.60  (51.49) | 1898.20  (51.46) |
| $\sigma_{g}^{2}$ | 1439.10 (72.37) | 1439.30 (72.38) | 1439.40 (72.38) | 1384.40  (71.60) | 1384.80  (71.55) |
| $\sigma_{e}^{2}$ | 1329.70 (610.22) | 970.82 (668.20) | 738.99 (540.02) | 981.58  (675.57) | 734.10  (540.91) |
| $\sigma_{\mathrm{ec}}^{2}$ | -- | 447.33 (868.51) | 712.24 (913.01) | 403.81  (839.97) | 710.99  (918.54) |
| $\sigma_{\mathrm{ge}}^{2}$ | -- | -- | -- | 169.95  (26.84) | 178.24  (27.14) |
| $\sigma_{\epsilon}^{2}$ | 4382.70 (51.80) | 4382.70 (40.69) | 4382.70 (40.69) | 4322.3  (41.23) | 4317.50  (41.24) |
| $h^{2}$ | 0.16  (0.01) | 0.16  (0.01) | 0.17  (0.02) | 0.16  (0.02) | 0.17  (0.01) |
| $e^{2}$ | 0.15  (0.06) | 0.10  (0.07) | 0.08  (0.06) | 0.11  (0.07) | 0.08  (0.06) |
| $\mathrm{ec}^{2}$ | -- | 0.05  (0.10) | 0.08  (0.11) | 0.05  (0.10) | 0.09  (0.11) |
| $\mathrm{ge}^{2}$ | -- | -- | -- | 0.02  (0.00) | 0.02  (0.00) |

^1^$\sigma_{l}^{2}$ = variance due to litter; $\sigma_{g}^{2}$ = additive genetic variance; $\sigma_{e}^{2}$ = environmental variance; $\sigma_{\mathrm{ce}}^{2}$ = environmental covariates variance; $\sigma_{\mathrm{ge}}^{2}$ = genotype by environmental interaction variance; $\sigma_{\epsilon}^{2}$ = residual variance; $h^{2}$ = heritability; $e^{2}$ = proportion of the phenotypic variance explained by environmental effect – environmentability; $\mathrm{ec}^{2}$ = proportion of the phenotypic variance explained by the environmental covariates; $\mathrm{ge}^{2}$ = proportion of phenotypic variance explained by the genotype by environmental interaction. Abbreviations: MG = traditional genomic best linear unbiased predictor (GBLUP) model; ME30 = GBLUP considering environmental effects correlated based on 30 days of weather information; ME100 = GBLUP considering environmental effects correlated based on 100 days of weather information; MGE30 = GBLUP considering genotype by environment interaction (GE) based on 30 days of weather information; MGE100 = GBLUP considering GE based on 100 days of weather information; ADG = average daily gain; and BFT = backfat thickness.

**Table S2 (continued) Variance components (SE), heritability (SE), and environmentability (SE) estimates, along with proportion of phenotypic variance due to genotype-by-environment interaction for all tested models including an extra uncorrelated environmental random effect for ME_30_, ME_100_, MGE_30_, and MGE_100_ according to the studied trait**

| **Variance Components^1^** | **Models** | | | | |
| --- | --- | --- | --- | --- | --- |
|  | **MG** | **ME_30_** | **ME_100_** | **MGE_30_** | **MGE_100_** |
| BFT | | | | | |
| $\sigma_{l}^{2}$ | 0.38  (0.01) | 0.38  (0.01) | 0.38  (0.01) | 0.38  (0.01) | 0.36  (0.01) |
| $\sigma_{g}^{2}$ | 0.84  (0.04) | 0.84  (0.01) | 0.84  (0.04) | 0.84  (0.04) | 0.79  (0.03) |
| $\sigma_{e}^{2}$ | 2.11  (0.95) | 2.11  (0.90) | 1.85  (1.01) | 2.11  (1.08) | 1.82  (1.00) |
| $\sigma_{\mathrm{ec}}^{2}$ | -- | 0.00  (0.00) | 0.23  (0.67) | 0.00  (0.50) | 0.24  (0.68) |
| $\sigma_{\mathrm{ge}}^{2}$ | -- | -- | -- | 0.00  (0.00) | 0.08  (0.02) |
| $\sigma_{\epsilon}^{2}$ | 1.51  (0.02) | 1.51  (0.01) | 1.51  (0.02) | 1.51  (0.02) | 1.48  (0.02) |
| $h^{2}$ | 0.17 (0.04) | 0.18  (0.04) | 0.18  (0.05) | 0.18  (0.06) | 0.19  (0.01) |
| $e^{2}$ | 0.44  (0.14) | 0.41  (0.12) | 0.34  (0.00) | 0.40  (0.18) | 0.37  (0.20) |
| $\mathrm{ec}^{2}$ | -- | 0.00  (0.00) | 0.05  (0.17) | 0.01  (0.12) | 0.08  (0.19) |
| $\mathrm{ge}^{2}$ | -- | -- | -- | 0.00  (0.00) | 0.02  (0.01) |

^1^$\sigma_{l}^{2}$ = variance due to litter; $\sigma_{g}^{2}$ = additive genetic variance; $\sigma_{e}^{2}$ = environmental variance; $\sigma_{\mathrm{ce}}^{2}$ = environmental covariates variance; $\sigma_{\mathrm{ge}}^{2}$ = genotype by environmental interaction variance; $\sigma_{\epsilon}^{2}$ = residual variance; $h^{2}$ = heritability; $e^{2}$ = proportion of the phenotypic variance explained by environmental effect – environmentability; $\mathrm{ec}^{2}$ = proportion of the phenotypic variance explained by the environmental covariates; $\mathrm{ge}^{2}$ = proportion of phenotypic variance explained by the genotype by environmental interaction. Abbreviations: MG = traditional genomic best linear unbiased predictor (GBLUP) model; ME30 = GBLUP considering environmental effects correlated based on 30 days of weather information; ME100 = GBLUP considering environmental effects correlated based on 100 days of weather information; MGE30 = GBLUP considering genotype by environment interaction (GE) based on 30 days of weather information; MGE100 = GBLUP considering GE based on 100 days of weather information; ADG = average daily gain; and BFT = backfat thickness.
