# Supplementary material for: Genotype-by-environment interaction with high-dimensional environmental data: an example in pigs
Source: Genet Sel Evol. 2025 Jun 5;57:28. doi: 10.1186/s12711-025-00974-2 (PMC12142960; doi:10.1186/s12711-025-00974-2)
Supplement: Supplementary file 3 — Additional file 3: Table S3. Model fit information for all tested models, including an extra uncorrelated environmental random effect for ME30, ME100, MGE30, and MGE100 according to the studied trait. Results contain estimated variance components for all models when an extra uncorrelated random environmental effect was included for ME30, ME100, MGE30, and MGE100. [file 12711_2025_974_MOESM3_ESM.docx]

**Table S3 Model fit information for all tested models including an extra uncorrelated environmental random effect for ME_30_, ME_100_, MGE_30_, and MGE_100_ according to the studied trait**

| **Model** | **-2LogL^1^** | **AIC^2^** | **BIC^3^** |
| --- | --- | --- | --- |
| ADG | | | |
| MG | 410,039.34 | 410,047.34 | 410,081.24 |
| ME_30_ | 410,039.30 | 410,049.30 | 410,102.15 |
| ME_100_ | 410,038.64 | 410,048.64 | 410,101.49 |
| MGE_30_ | 409,965.95 | 409,977.95 | 410,028.80 |
| MGE_100_ | 409,957.81 | 409,969.81 | 410,020.66 |
| BFT | | | |
| MG | 114,144.04 | 114,152.04 | 114,185.52 |
| ME_30_ | 114,144.04 | 114,154.04 | 114,195.89 |
| ME_100_ | 114,143.83 | 114,153.83 | 114,195.68 |
| MGE_30_ | 114,142.04 | 114,154.04 | 114,204.26 |
| MGE_100_ | 114,041.63 | 114,053.63 | 114,103.85 |

^1^Log likelihood function; ^2^Akaike information criteria; and ^3^Bayesian information criteria. Abbreviations: MG = traditional genomic best linear unbiased predictor (GBLUP) model; ME_30_ = GBLUP considering environmental effects correlated based on 30 days of weather information; ME_100_ = GBLUP considering environmental effects correlated based on 100 days of weather information; MGE_30_ = GBLUP considering genotype by environment interaction (GE) based on 30 days of weather information; MGE_100_ = GBLUP considering GE based on 100 days of weather information; MTM = multiple trait GBLUP model; ADG = average daily gain; and BFT = backfat thickness.
